# *O*-linked *N*-acetylglucosamine transferase (OGT) is overexpressed and promotes *O*-linked protein glycosylation in esophageal squamous cell carcinoma

**DOI:** 10.7555/JBR.26.20110121

**Published:** 2012-06-25

**Authors:** Zhe Qiao, Chengxue Dang, Bin Zhou, Shaomin Li, Wei Zhang, Jiantao Jiang, Jin Zhang, Ranran Kong, Yuefeng Ma

**Affiliations:** a Department of Thoracic Surgery, the Second Affiliated Hospital, Xi'an Jiaotong University, Xi'an, Shaanxi 710004, China;; b Department of Surgical Oncology, the First Affiliated Hospital, Xi'an Jiaotong University, Xi'an, Shaanxi 710061, China.

**Keywords:** *O*-linked *N*-acetylglucosamine (*O*-GlcNAc) transferase (OGT), *O*-linked glycosylation (*O*-GlcNAcation), esophageal squamous cell carcinoma

## Abstract

The aim of this present study was to explore the expression and clinical significance of *O*-linked *N*-acetylglucosamine (*O*-GlcNAc) transferase (OGT) and enzymatic *O*-linked glycosylation (*O*-GlcNAcation) through the addition of *O*-linked-β-*N*-acetylglucosamine in esophageal squamous cell carcinoma. OGT expression and *O*-GlcNAcation in 40 samples from patients with esophageal squamous cell carcinoma was detected by immunohistochemical staining with anti-OGT antib ody and *O*-GlcNAc-specific antibody RL_2_, respectively. The relationship between pathological and clinical factors of patients was analyzed. We found that the expression of OGT was higher in esophageal squamous cell carcinoma samples compared to the normal tissues. RL_2_ antibody level was positively correlated with OGT expression, and the metastasis of lymph node, which means the level of *O*-GlcNAcation was high and related to the metastasis of lymph node in esophageal squamous cell carcinoma. In conclusion, OGT activation is the main reason for promoting the level of *O*-GlcNAcation in esophageal squamous cell carcinoma. *O*-GlcNAcylation may play an important role in esophageal squamous cell carcinoma.

## INTRODUCTION

Cancer cells upregulate glycolysis and increase glucose uptake to meet energy needs. A small fraction of glucose in the cell enters the hexosamine biosynthetic pathway (HBP)[1]–[3], which regulates enzymatic *O*-linked glycosylation (*O*-GlcNAcation), a carbohydrate posttranslational modification of diverse nuclear and cytosolic proteins by the addition of *O*-linked-β-*N*-acetylglucosamine(O-GlcNAc). *O*-GlcNAcation participates in the modification of protein and plays an important role in oncogenesis and progression of tumor and signal transduction[4]–[6]. GlcNAcylation plays a role in normal biological processes, and its dysregulation is involved in certain human diseases such as diabetes mellitus[7],[8] and neurologic disorders[9]. In recent reports, several tumor-associated proteins have been identified as GlcNAcylated proteins, including c-myc[10] and p53[11]. We hypothesized that *O*-GlcNAcylation participates in tumor occurrence. In this present study, we explored the expression of *O*-GlcNAc transferase (OGT) and *O*-GlcNAcation by immunohistochemistry using an *O*-GlcNAc-specific antibody-RL_2_ in esophageal squamous cell carcinoma. We found that the levels of both OGT and RL_2_ were high in esophageal squamous cell carcinoma, suggesting that *O*-GlcNAcylation may play an important role in esophageal squamous cell carcinoma.

## SUBJECTS AND METHODS

### Subjects

We recruited 40 patients with esophageal squamous cell carcinoma, who had undergone esophagectomy between 2007 and 2009 at the Department of Thoracic Surgery, the Second Affiliated Hospital of Xi'an Jiaotong University, Xi'an, Shaanxi, China. Normal esophageal mucosa was got from the same patient (after the operation the esophageal mucosa was confirmed normal by pathological examination). Cancer was staged according to the 2009 UICC TNM classification. The study protocol was approved by the local Institutional Ethics Committee, and written informed consent was obtained from all the patients.

### Reagents

Rabbit polyclonal anti-OGT was bought from Protein Tech Group (Chicago, IL, USA). Mouse monoclonal anti-*O*-linked *N*-acetylglucosamine antibody (RL_2_) was bought from Thermo Scientific (Rockford, IL, USA).

### Immunohistochemistry

Tissues were fixed in 40 g/L paraformaldehyde, embedded in paraffin, and sectioned at a thickness of 5 µm. Sections were dewaxed, dehydrated, and then stained by SP kit (Zhongshan Goldenbridge Biotechnology Co. Ltd., Beijing, China). Antibodies of OGT and RL_2_ were diluted to a final concentration of 1:100 with TBS. OGT positive pancreatic cancer samples and RL_2_ positive gastric cancer samples were used as positive controls. Positivity of OGT or RL_2_ was considered when yellow or brown yellow granules appeared in the nucleus and cytoplasm, respectively. Samples were considered positive when more than 5% of tumor cells were stained positive, and strongly positive when more than 50% of tumor cells were stained positive. Otherwise, it was considered low expression. The positive extent was judged through density by ImagePro6.0 (Media Cybernetics, Bethesda, MD, USA), which could analyze the correlation between the expression of OGT and RL_2_.

### Statistical analysis

SPSS 13.0 software (SPSS Inc., Chicago, IL, USA) was used to analyze the details. The Fish's exact test was used to analyze the levels of OGT and RL_2_. The correlation analysis was used for the evaluation of the relationship between OGT and RL_2_. *P* values less than 0.05 were considered to be significant.

## RESULTS

### The demographic characteristics of subjects

Forty esophageal squamous cell carcinoma patients were recruited including 28 males and 12 females. Age ranged from 40 to 77 years and the mean age was 64.6 years. None of the patients received neoadjuvant chemotherapy or radiotherapy before esophagectomy. The clinical stages were from T_2_ to T_3_ and lymph node metastasis was local. There were no distant metastatic cases.

### Expression of OGT and *O*-GlcNAcation in esophageal squamous cell carcinoma

**Fig. 1:**
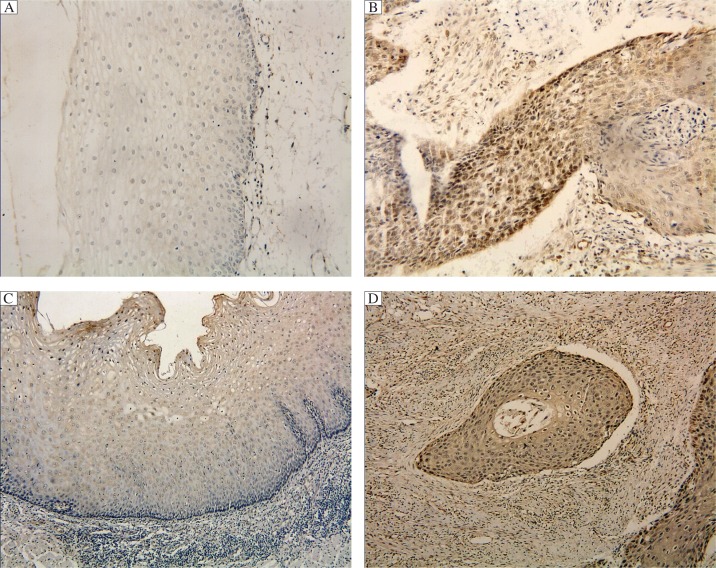
OGT and RL_2_ expression in normal and esophageal squamous cell carcinoma (SP staining, ×100). A: Expression of OGT in normal esophageal mucosa. B: Expression of OGT in esophageal squamous cell carcinoma. C: Expression of *O*-GlcNAc in normal esophageal mucosa. D: Expression of *O*-GlcNAc in esophageal squamous cell carcinoma.

The levels of OGT and *O*-GlcNAcation in esophageal squamous cell carcinoma and normal esophageal mucosa were detected by immunohistochemistry using anti-OGT antibody and RL_2_ antibody, respectively. Most normal esophageal tissues (30/35, 85.7%) showed low expression of OGT while esophageal squamous cell carcinoma tissues mostly (35/45, 77.8%) exhibited high expression (*P* < 0.01, ***Fig. 1A*** and ***1B***, ***Table 1***). Tumor size was not correlated with OGT expression (*P* = 0.78, ***Table 1***). The difference in the expression of OGT between T2 and T3 stage tumors was not significant (*P* = 1.0, ***Table 1***). The expression of OGT in metastatic lymph nodes or non-metastatic lymph nodes was not different (*P* = 0.627, ***Table 1***). Gender and age were not correlated with OGT (*P* > 0.05 in both, ***Table 1***).

RL_2_ was positive in normal esophageal tissue, but strongly positive in esophageal squamous cell carcinoma tissues (*P* = 0.02, ***Fig. 1C*** and ***1D***, ***Table 2***). The samples with lymph node metastasis showed higher RL_2_ expression compared to samples without lymph node metastasis (*P* = 0.046, ***Table 2***). Tumor size, T stage, gender and age were not correlated with RL_2_ positive status (*P* > 0.05 in all, ***Table 2***). According to the optical density analyzed by ImagePro6.0, in samples with lymph node metastasis, the optical density of OGT was 250.3±16.28, and that of RL_2_ was 171.5±16.18. The density of RL_2_ was positively correlated with OGT (*r* = 0.996, *P* = 0.028).

**Table 1:** Relationship between OGT expression and pathologic features of esophageal cancer patients

Pathological factor	Low expression of OGT	High expression of OGT	*P* value
Sample source			
Esophageal cancer	5	35	0
Normal esophageal mucosa	30	10	
T stage			
T2	1	20	1.0
T3	4	15	
Length of cancer			
> 5 cm	2	22	0.78
< 5 cm	3	13	
Lymph node metastasis			
(+)	3	25	0.627
(–)	2	10	
Gender			
Male	3	25	0.627
Female	2	10	
Age (years)			
< 50	1	18	0.345
> 50	4	17	

(*n* = 40)

**Table 2:** Relationship between RL_2_ level and pathologic features in esophageal cancer patients

Pathological factor	Low expression of RL_2_	High expression of RL_2_	*P* value
Sample source			
Esophageal cancer	10	30	0.02
Normal esophageal mucosa	25	15	
T stage			
T2	7	14	0.181
T3	3	16	
Length of cancer			
> 5 cm	4	20	0.159
< 5 cm	6	10	
Lymph node metastasis			
(+)	5	23	0.046
(–)	6	6	
Gender			
Male	6	22	0.451
Female	4	8	
Age (years)			
< 50	4	15	0.721
> 50	6	15	

(*n* = 20)

## DISCUSSION

Protein modification includes phosphorylation, glycosylation, ubiquitination, glycosylation, nitrolation, and *O*-GlcNAcation. *O*-GlcNAcation is a kind of carbohydrate posttranslational modification of diverse nuclear and cytosolic proteins, which is the post-translational cycling of a single *O*-linked-β-*N*-acetylglucosamine on the hydroxyl groups of serine and/or threonine residues of target proteins[5],[12],[13]. O-GlcNAcylation is dynamically regulated by OGT and β-N-acetylglucosaminidase (OGA). OGT (polypeptide β-N-acetylglucosaminyltransferase) catalyzes the addition of O-linked-β-N-acetylglucosamine from UDP-GlcNAc onto the hydroxyl group of a serine or a threonine residue on the protein substrates[14]. OGA is a neutral hexosaminidase with a catalytic site similar to that of the family 84 glycoside hydrolase that specifically catalyzes the removal of β-linked GlcNAc on its substrate[15],[16],[17].

When *O*-GlcNAcation modifies the protein, it can regulate phosphorylation of substrates at the same time, so there should be some relationships between phosphorylation and *O*-GlcNAcation[18]. A study showed that *O*-GlcNAcation competed with phosphorylation[19]. In fact, most of cellular signal transduction was related to the phosphorylation of proteins, so the level of cellular *O*-GlcNAcation may affect cellular signal transduction. Abnormal cellular signal transduction is one of the reasons for the occurrence and development of cancer.

Our study showed that OGT expression and *O*-GlcNAcation level were both increased in esophageal squamous cell carcinoma tissues compared to normal esophageal mucosa tissues (*P* < 0.05). Similar results have been reported in lung and colon cancer[20]. *O*-GlcNAcation of protein usually competes with phosphorylation of protein[21]. As long as the protein is GlcNAcylated, protein degradation through the phosphorylation pathway is inhibited, and then GlcNAcylated protein becomes stable. Some oncoproteins, such as p53 and c-myc, which also can be GlcNAcylated, become stable after GlcNAcylation and are increased inside the cells[22],[23], and then activate abnormal cellular signal transduction[23],[24],[25]. In hepatocellular carcinoma followed by liver transplantation, O-GlcNAcylation was significantly enhanced in the tumor tissues of patients, who suffered from recurrence of hepatocellular carcinoma[26], suggesting that the level of O-GlcNAcation in hepatocellular carcinoma might be related to tumor reccurrence. In our study, the length, age, gender and T stage of cancer did not affect the levels of OGT and O-GlcNAcation (P > 0.05, ***Table 1***, 2). These results support the opinion that the level of O-GlcNAcation might be related to the cancerous occurrence.

In fact, *O*-GlcNAcation is regulated by OGT and OGA. Slawson *et al*.[28] found that the activity of OGA was higher in breast cancer, especially in more aggressive tumors. By contrast, tumor cell lines that mimic metastatic tumors showed an increase in OGT expression and *O*-GlcNAcation, suggesting that an increase in *O*-GlcNAcylation may be beneficial to cancer cells[29]. So there is a conflict between OGT and OGA in breast cancer. We also investigated the expression of OGA in esophageal squamous cell carcinoma, but the result shows that there is no difference between expression in normal esophageal tissue and esophageal squamous cell carcinoma. Our statistical analysis showed that OGT was highly expressed in esophageal squamous cell carcinoma, and was positively correlated with *O*-GlcNAcation (*r* = 0.996, *P* = 0.028). The results indicated that OGT promoted *O*-GlcNAcation and played a more important role than OGA in esophageal squamous cell carcinoma, suggesting that in esophageal squamous cell carcinoma, OGT was the main protein responsible for *O*-GlcNAcation. In fact, high expression of OGT not only promotes the level of cellular *O*-GlcNAcation, but also affects cancerous occurrence. The TPR domain of OGT could promote protein interactions and affect the function of spindle[30]. This process produces many aneuploid cells, which result in tumor occurrence[31].

In our study, RL_2_ is strongly positive in the cancer samples with lymph node metastasis compared with those with no lymph node metastasis (*r* = 0.996, *P* = 0.046), suggesting that the high level of *O*-GlcNAcation is conducive to the metastasis of esophageal squamous cell carcinoma, especially to lymph nodes. This phenomenon has been observed in breast cancer[32]. Gu found that GlcNAcylated p120 and β-catenin affect the metastasis of breast cancer, especially in lymph node metastasis^[33]^.

In brief, based on our study, we concluded that high expression of OGT promoted the increase of *O*-GlcNAcation in esophageal squamous cell carcinoma and the high level of *O*-GlcNAcation may stabilize proteins, leading to changes in cellular signal transduction and resulting tumorigenesis and metastasis. Decreasing *O*-GlcNAcation by inhibiting OGT may benefit treatment of esophageal squamous cell carcinoma.

## References

[1] Zeidan Q, Hart GW (2010). The intersections between O-GlcNAcylation and phosphorylation: implications for multiple signaling pathways. J Cell Sci.

[2] Marshall S, Bacote V, Traxinger RR (1991). Role of hexosamine biosynthesis in the induction of insulin resistance. J Biol Chem.

[3] Copeland RJ, Bullen JW, Hart GW (2008). Cross-talk between GlcNAcylation and phosphorylation: roles in insulin resistance and glucose toxicity. Am J Physiol Endocrinol Metab.

[4] Barone BB, Yeh HC, Snyder CF, Peairs KS, Stein KB, Derr RL (2008). Long-term all-cause mortality in cancer patients with preexisting diabetes mellitus: a systematic review and meta-analysis. J Am Med Assoc.

[5] Torres CR, Hart GW (1984). Topography and polypeptide distribution of terminal N-acetylglucosamine residues on thesurfaces of intact lymphocytes. Evidence for O-linked GlcNAc. J Biol Chem.

[6] Hart GW, Housley MP, Slawson C (2007). Cycling of O-linked β-N-acetylglucosamine on nucleocytoplasmic proteins. Nature.

[7] Housley MP, Rodgers JT, Udeshi ND, Kelly TJ, Shabanowitz J, Hunt DF (2008). O-GlcNAc regulates Foxo activation in response to glucose. J Biol Chem.

[8] Vosseller K, Wells L, Lane MD, Hart GW (2002). Elevated nucleocytoplasmic glycosylation by O-GlcNAc results in insulin resistance associated with defects in Aktactivation in 3T3-L1 adipocytes. Proc Natl Acad Sci.

[9] Yang JY, Gu JL, Shi JH, Liu F, Shen Q (2008). Inhibitory effect of OGT gene expression on the level of tau phosphorylation. Jiangsu Daxue Xuebao (Medicine Edition) (in Chinese).

[10] Vervoorts J, Lüscher-Firzlaff J, Lüscher B (2006). The ins and outs of MYC regulation by posttranslational mechanisms. J Biol Chem.

[11] Yang WH, Kim JE, Nam HW, Ju JW, Kim HS, Kim YS (2006). Modification of p53 with O-linked Nacetylglucosamine regulates p53 activity and stability. Nat Cell Biol.

[12] Hart GW, Housley MP, Slawson C (2007). Cycling of O-linked beta-N-acetylglucosamine on nucleocytoplasmic proteins. Nature.

[13] Love DC, Hanover JA (2005). The hexosamine signaling pathway: deciphering the “O-GlcNAc code”. Sci STKE.

[14] Haltiwanger RS, Holt GD, Hart GW (1990). Enzymatic addition of O-GlcNAc to nuclear and cytoplasmic proteins. Identification of a undine diphospho-A-acetylglucosamine: peptide B-N-acetylglucosaminyltransferase. J Biol Chem.

[15] Dong DL, Hart GW (1994). Purification and characterization of an O-GlcNAc selective N-acetyl-B-D-glucosaminidase from rat spleen cytosol. J Biol Chem.

[16] Cetinbas N, Macauley MS, Stubbs KA, Drapala R, Vocadlo DJ (2006). Identification of asp174 and asp175 as the key catalytic residues of human o-glcnacase by functional analysis of site-directed mutants. Biochemistry.

[17] Gao Y, Wells L, Comer FI, Parker GJ, Hart GW (2001). Dynamic O-glycosylation of nuclear and cytosolic proteins: cloning and characterization of a neutral, cytosolic b-N-acetylglucosaminidase from human brain. J Biol Chem.

[18] Wells L, Kreppel LK, Comer FI, Wadzinski BE, Hart GW (2004). O-GlcNAc transferase is in a functional complex with protein phosphatase 1 catalytic subunits. J Biol Chem.

[19] Kang ES, Han D, Park J, Kwak TK, Oh MA, Lee SA (2008). O-GlcNAc modulation at Akt1 Ser473 correlates with apoptosis of murine pancreatic beta cells. Exp Cell Res.

[20] Mi W, Gu Y, Han C, Liu H, Fan Q, Zhang X (2011). O-GlcNAcylation is a novel regulator of lung and colon cancer malignancy. Biochim Biophys Acta.

[21] Holt GD, Snow CM, Senior A, Haltiwanger RS, Gerace L, Hart GW (1987). Nuclear port complex glycoproteins contain cytoplasmically disposed O-linked N-acetylglucosamine. J cell Biol.

[22] Jones SN, Roe AE, Donehower LA, Bradley A (1995). Rescue of embryonic lethality in Mdm2-deficient mice by absence of p53. Nature.

[23] Chou TY, Hart GW, Dang CV (1995). c-Myc is glycosylated at threonine 58, a known phosphorylation site and a mutational hot spot in lymphomas. J Biol Chem.

[24] Chou TY, Dang CV, Hart GW (1995). Glycosylation of the c-Myc transactivation domain. Proc Natl Acad Sci USA.

[25] Jörg Vervoorts, Juliane Lüscher-Firzlaff, Bernhard Lüscher (2006). The ins and outs of MYC regulation by posttranslational mechanisms. J Biol Chem.

[26] Yang WH, Kim JE, Nam HW, Ju JW, Kim HS, Kim YS (2006). Modification of p53 with O-linked Nacetylglucosamine regulates p53 activity and stability. Nat Cell Biol.

[27] Zhu QQ, Zhou L, Yang Z, Lai MC, Xie HY, Wu LM (2012). O-GlcNAcylation plays a role in tumor recurrence of hepatocellular carcinoma following liver transplantation. Med Oncol.

[28] Slawson C, Pidala J, Potter R (2001). Increased N-acetyl-beta-glucosaminidase activity in primary breast carcinomas corresponds to a decrease inN-acetylglucosamine containing proteins. Biochim Biophys Acta.

[29] Caldwell SA, Jackson SR, Shahriari KS, Lynch TP, Sethi G, Walker S (2010). Nutrient sensor O-GlcNAc transferase regulates breast cancer tumorigenesis through targeting of the oncogenic transcription factor FoxM1. Oncogene.

[30] Slawson C, Zachara NE, Vosseller K, Cheung WD, Lane MD, Hart GW (2005). Perturbations in O-linked beta-Nacetylglucosamine protein modification cause severe defects in mitotic progression and cytokinesis. J Biol Chem.

[31] Bannon JH, Mc Gee MM (2009). Understanding the role of aneuploidy in tumorigenesis. Biochem Soc Trans.

[32] Gu Y, Mi W, Ge Y, Liu H, Fan Q, Han C (2010). GlcNAcylation Plays an Essential Role in Breast Cancer Metastasis. Cancer Res.

